# Low Immune Response to Hepatitis B Vaccine among Children in Dakar, Senegal

**DOI:** 10.1371/journal.pone.0038153

**Published:** 2012-05-30

**Authors:** Marie-Anne Rey-Cuille, Abdoulaye Seck, Richard Njouom, Loïc Chartier, Housseyn Dembel Sow, Mamadou, Amadou Sidy Ka, Mohamadou Njankouo, Dominique Rousset, Tamara Giles-Vernick, Guillemette Unal, Jean-Marie Sire, Benoît Garin, François Simon, Muriel Vray

**Affiliations:** 1 Unité de Recherche et d'Expertise en Epidémiologie des Maladies Emergentes, Institut Pasteur, Paris, France; 2 Institut des Sciences Biologiques, CNRS, Paris, France; 3 Laboratoire de Biologie Médicale, Institut Pasteur, Dakar, Sénégal; 4 Service de Virologie, Centre Pasteur du Cameroun, Yaoundé, Cameroun; 5 Hôpital d'Enfants Albert Royer, Dakar, Sénégal; 6 Service de Pédiatrie, Hôpital Principal, Dakar, Sénégal; 7 Département de virologie, Hôpital Saint Louis, Paris, France; 8 Institut National de la Santé et de la Recherche Médicale (INSERM), Paris, France; Centers for Disease Control and Prevention, United States of America

## Abstract

HBV vaccine was introduced into the Expanded Programme on Immunization (EPI) in Senegal and Cameroon in 2005. We conducted a cross-sectional study in both countries to assess the HBV immune protection among children. All consecutive children under 4 years old, hospitalized for any reason between May 2009 and May 2010, with an immunisation card and a complete HBV vaccination, were tested for anti-HBs and anti-HBc. A total of 242 anti-HBc-negative children (128 in Cameroon and 114 in Senegal) were considered in the analysis. The prevalence of children with anti-HBs ≥10 IU/L was higher in Cameroon with 92% (95% CI: 87%–97%) compared to Senegal with 58% (95% CI: 49%–67%), (p<0.001). The response to vaccination in Senegal was lower in 2006–2007 (43%) than in 2008–2009 (65%), (p = 0.028). Our results, although not based on a representative sample of Senegalese or Cameroonian child populations, reveal a significant problem in vaccine response in Senegal. This response problem extends well beyond hepatitis B: the same children who have not developed an immune response to the HBV vaccine are also at risk for diphtheria, tetanus, pertussis (DTwP) and *Haemophilus influenzae* type b (Hib). Field biological monitoring should be carried out regularly in resource-poor countries to check quality of the vaccine administered.

## Introduction

The prevalence of HBV chronic infection is particularly high in Sub Saharan Africa, ranging from 7 to 26% [1]. Because available treatment for hepatitis B virus infection does not provide a complete cure and is very costly in developing countries, prevention remains crucial [2]. Previous studies have demonstrated immunogenicity of HBV vaccine ranging between 88% and 94% with anti-HBs response ≥10 mUI/ml, associated (pentavalent vaccine) or not with diphtheria-tetanus-pertussis (DTwP) and *Haemophilus influenzae* type b (Hib) [3], [4], [5], [6]. In 1991, the World Health Organization recommended that all countries introduce hepatitis B vaccination into their routine national infant immunisation programmes [7].

In Senegal, previous studies have demonstrated an HBV chronic infection prevalence of 17% among blood donors [8], a prevalence of HBV exposure of 60% among children between 0 and 5 years old [9], and 14 % of HBsAg positivity among pregnant women [10]. In Cameroon, the prevalence of HBsAg varies from 12% among Pygmies [11] to 20% among children of primary school age [12] and 25 % among a population older than 4 years of age [13]. In response to WHO recommendations, Cameroon and Senegal integrated the HBV vaccination into the Expanded Programme on Immunization (EPI) in 2005. The DTwP-HBV-Hib combination vaccine Zilbrix™ developed by GSK is currently used by the National Programme of Cameroon, while Quinvaxem^TM^, a DTwP-HBV-Hib vaccine co-developed by Crucell and Novartis, is administrated in Senegal. Both combination vaccines have been shown to be immunogenic and well tolerated [5], [14]. The three doses of the pentavalent vaccine are administrated at 6, 10 and 14 weeks of age in both countries, according to WHO recommendations. With the introduction and expansion of the HBV vaccine in sub-Saharan Africa, it remains imperative to monitor the seroprotective response to HBV childhood immunisation programmes.

We conducted a cross-sectional study in Senegal and Cameroon in order to assess the response to vaccination among children with a complete HBV vaccination.

## Results

A total of 242 children with an immunisation card were recruited, 128 in Cameroon, and 114 in Senegal (Table 1). Forty-seven percent of children were female, and the overall median age was 15 months [9]; [21]. Senegal presented a higher prevalence of children suffering from moderate or severe malnutrition (66%), compared to Cameroon (12%), (p<0.001). The main causes of the children's hospitalization were gastro-intestinal infections and infectious syndromes in Cameroon (60%), and gastro-intestinal infections and respiratory diseases in Senegal (60%). The median [IQR] delays between the first and the second dose of the HBV vaccination and between the second and the third dose were 30 days [28; 35] and 31 days [28; 35] in Cameroon, and 32 days [30; 35] and 33 days [31; 36] in Senegal, respectively. The prevalence of children with anti-HBs ≥10 IU/L was higher in Cameroon, with 92% (95% CI: 87%–97%) compared to Senegal, with 58% (95% CI: 49%–67%), (p<0.001). The proportion of children with anti-HBs ≥100 IU/L was 66% and 23% in Cameroon and Senegal, respectively (p<0.001) (Table 1).

**Table 1:** Characteristics of the children.

Variables	Cameroon N = 128	Senegal N = 114	Total N = 242	p
**Sex**, female N (%)	55(43)	59 (52)	114 (47)	0.17
**Age** (months)*	13 [9]; [20]	17 [11; 24]	15 [9]; [21]	0.004
**Moderate or severe malnutrition **(**WAZ-score ≤−2**) N *(%)*	**15** (**12**)	**75** (**66**)	**90** (**38**)	<0.001
**Reasons for hospitalisation** N (%)				<0.001
Gastro-intestinal infection	34 (27)	33 (29)	67 (28)	
Respiratory Infection	19 (15)	35 (31)	54 (22)	
Malaria	16 (13)	2 (2)	18 (7)	
Other Infectious Syndrome	**42** (**33**)	13 (11)	55 (23)	
Other reasons (convulsions, anaemia, malnutrition)	17 (13)	**31** (**27**)	48 (20)	
**Delay of the HBV vaccination** (**days**)*				
Between the first and the second dose	30 [28; 35]	32 [30; 35]	32 [28;35]	0.001
Between the second and the third dose	31 [28; 35]	33 [31; 36]	32 [29; 35]	0.008
**Anti-HBs** N (%)				
** ≥10 IU/L**	118 (92)	66 (58)	184 (76)	<0.001
** ≥100 IU/L**	85 (66)	26 (23)	111 (46)	<0.001

* ***Median [Q1; Q3]***.

In the two countries, the children were recruited in two hospitals and no differences were revealed in the percentages of responders. The Cameroon hospitals showed 95% (Essos Hospital) versus 87% (Chantal Biya Foundation) responding (p = 0.17), whereas the Senegal hospitals indicated 56% (Albert Royer Paediatric Hospital) versus 65% (Hôpital Principal) responding (p = 0.48) (data not shown).

Taking into account the year of vaccination, we found that in Senegal, the response to vaccination was lower in 2006–2007 (43%), compared with 2008–2009 (65%), (p = 0.028).

**Figure 1:**
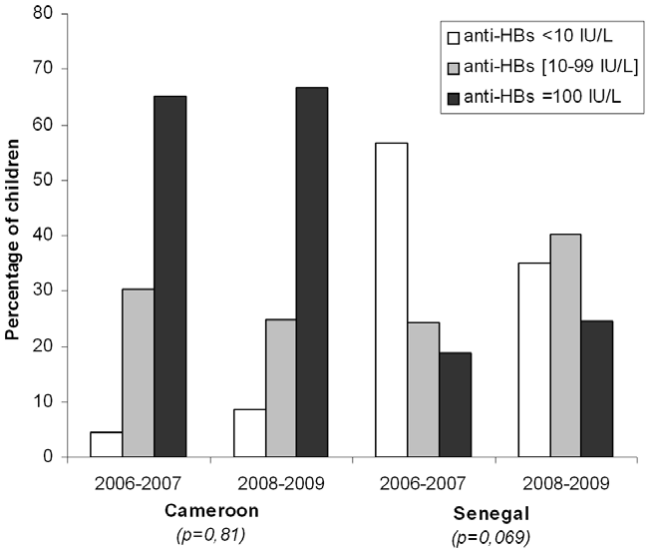
Immune response (anti-HBs levels) to complete HBV vaccination according to the year of vaccination in Cameroon and Senegal among children with immunization card.

In contrast, in Cameroon, no difference existed between the prevalence of children anti-HBs+ during these two time periods (96% versus 91%, p = 0.69) (Figure 1).

Nutritional status was significantly correlated with response to the HBV vaccination (p<0.001), with 85% of children protected (anti-HBs ≥10 IU/L) among those with normal nutrition status versus 60 % of children with moderate to severe malnutrition. The percentages of protected children were lower in the two countries among the children with moderate or severe malnutrition (12% vs 20% in Cameroon, 62% vs 71% in Senegal). However, the nutritional status was not significant when adjusted for the country of residence (Table 2).

**Table 2:** Factors associated with HBV vaccine response.

	Anti-HBs		Univariate analysis		Multivariate analysis	
**Variables**	**<10 IU/L N = 58**	**≥10 IU/L N = 184**	**OR** (**CI_95%_**_)_	**p**	**OR** (**CI_95%_**_)_	**p**
**Country** N (%)						
Cameroon	10 (17)	118 (64)	1		1	
Senegal	48 (83)	66 (36)	0.1 (0.06–0.2)	<0.001	0.1 (0.06–0.3)	<0.001
**Sex**, female N (%)	23 (40)	91 (49)	1.5 (0.8–2.7)	0.19		
**Age** ≤15 months N (%)	17 (29)	108 (59)	3.4 (1.8–6.5)	<0.001	3.1 (1.6–6.1)	0.001
**Moderate or severe malnutrition** N (%)	36 (62)	54 (31)	0.3 (0.1–0.5)	<0.001		

## Discussion

Overall, we note that the immunization schedule was well respected for all children in both countries, with a median time between doses close to 30 days.

While we observed in Cameroon a protection rate of 92%, corresponding with previously reported protection rates for hepatitis B vaccination in both developing and developed countries [3], [4], [5], we were surprised by the low protection level (58%) in Senegal. Moreover, in Senegal, the proportion of children responding to the vaccine varied dramatically over time with a low protected rate in 2006–2007 (43%).

Faced with these unexpected results, we sought to verify the hypothesis of lower vaccine immunogenicity, by measuring the response to the diphtheria antigens associated in the same vial. Antibodies against diphtheria antigens persist several months after vaccination [15]. In the present study, the detection of antibodies was performed using EIA (IgG testkit, Genzyme, Germany) in nineteen blood samples from Senegalese children vaccinated less than one year after vaccination: nine responder children (anti-HBs+) and ten vaccinated and non-responder children (anti-HBs–). Low anti-diphtheria antibody response was significantly associated with the lack of anti-HBs antibodies (p = 0.036), but the low value of correlation (r = 0.48) impaired any definitive conclusion. The low value of the coefficient between anti-diphtheria and anti-HBs antibodies is probably due to the small number of cases.

Several possible explanations may account for these results. First, there may exist problems with storage conditions in Senegal, since frequent power outages may provoke lapses in backup electrical systems and compromise the cold chain [16]. Second, there may be a quality problem with the pentavalent Quinvaxem^TM^ (Crucell) vaccine. In 2011, for instance, Quinvaxem production temporarily ceased because of sterilisation problems [17]. A third explanation may be related to children's nutritional status, which is much more severe among Senegalese children than among Cameroonian children. The small number of children with moderate or severe malnutrition in our study, especially in Cameroon, led to the result that when both variables (nutritional status and country) were introduced simultaneously in a multivariate model, only country remained significantly associated with antibody response. However, the percentages of protected children were lower in the two countries among those children with moderate or severe malnutrition. These results are in accordance with recent studies that reported no immune response difference between healthy children and those with compromised nutritional status [18], [19], [20], [21].

Although we cannot definitively explain the reason(s) for anti-HBV vaccination failure in Senegal, the striking disparity between our results, based on anti-HBs antibody levels, and vaccination card registrations demonstrates a critical need for monitoring accurately vaccine delivery and coverage. Current vaccination coverage surveys are based mainly on an assessment of immunisation cards [22]. Yet our results, although not based on a representative sample of Senegalese or Cameroonian child populations, reveal a significant problem in vaccine response in Senegal that present official surveys cannot detect. This response problem may extend well beyond hepatitis B: the same children who have not developed an immune response to the HBV vaccine are also at risk for diphtheria, tetanus, pertussis and *Haemophilus influenzae* B.

Current evaluations of vaccination programmes, particularly in resource-poor countries, necessitate supplemental and regular biological monitoring, to ensure vaccine quality and storage and to verify that vaccine recipients are genuinely protected. We would also call for further studies on larger populations in countries that participate in the EPI, so as to investigate more fully the vaccines and their storage and delivery. These results make evidence the need for collaboration between the Expanded Programme on Immunization and national programmes to control Hepatitis B. Such measures should constitute a clear global public health priority.

## Materials and Methods

### Study population

This study took place from May 2009 to May 2010, in two hospitals in Yaounde (Cameroon), the Essos Hospital and the Chantal Biya Foundation, and in two hospitals in Dakar (Senegal), the Hôpital Principal and the Albert Royer Paediatric Hospital.

All consecutive children, under 4 years old, hospitalized for any reason, with a blood sample prescribed during hospitalisation, sufficiently healthy to withstand an extended blood sample of 2 ml minimum, with an immunisation card and a complete HBV vaccination including the three injections according to the recommended schedule (first dose 6 weeks after birth and intervals between 2 injections of 30 days minimum) and anti-HBc-negative were considered in this analysis.

### Ethical approvals

Approval to conduct the study was obtained from the National Ethics Committee and the Ministry of Public Health of Cameroon and the National Comity of the Research in Health of Senegal. All children anti-HBs-negative and anti-HBc-negative were invited to return to receive an HBV vaccination free of charge, no matter the HBV vaccination status reported on their immunization card.

### Data collection

Demographic and socioeconomic characteristics (age, sex, weight, and reason for hospitalization), vaccination records and all serological data were collected through a structured questionnaire.

### Anthropometric measurements

Malnutrition status was estimated by the weight-for-age Z-score (WAZ), because most values for the children's height were missing. WAZ was calculated using the Centers for Disease Control and Prevention 2000 child growth charts (CDC-2000) [23]. Moderate or severe malnutrition was defined when the WAZ value was less or equal to −2.

### HBV markers

All samples were tested for anti-HBs and anti-HBc by enzyme immunoassay (EIA) (DiaSorin Biomedica, Sallugia, Italy). Anti-HBs antibodies were expressed in international units (IU/L). The level of anti-HBs of 10 IU/L and higher was considered to be seroprotective. The responders were defined as children anti-HBs+ and anti-HBc−, while the non responders were defined as children anti-HBs− and anti-HBc−.

### Diphtheria antibodies

The detection of antibodies against diphtheria was performed using EIA (IgG testkit, Genzyme, Germany). Levels of anti-diphtheria antibodies were expressed in international units (IU/ml). All sera with a level of anti-diphtheria of 0.1 UI/ml and higher were considered to be positive.

### Statistical analysis

Quantitative variables were expressed as median [Q1–Q3] and qualitative as percentages.

Anti-HBs antibodies were analysed using three categories: <10 IU/L, 10–99 IU/L, and ≥100 IU/L. The HBV immune response was measured taking into account the time of vaccination, 2006–2007 or 2008–2009, in the two countries. Univariate analysis was based on the chi2 test or Fisher's exact test for discrete variables and the Mann-Whitney test for continuous variables. For the multivariate analysis, quantitative variables were categorized around the median. All baseline variables associated with vaccine response in the univariate analysis (p≤0.25) were included in a backward stepwise logistic regression model. Interactions between factors associated with vaccine response and participating country (Senegal and Cameroon) were tested. Results were considered statistically significant when p<0.05. Correlation between anti-diphtheria antibody response and anti-HBs antibodies was based on the Spearman coefficient and linear regression. STATA 11 software was used for all statistical analyses.
